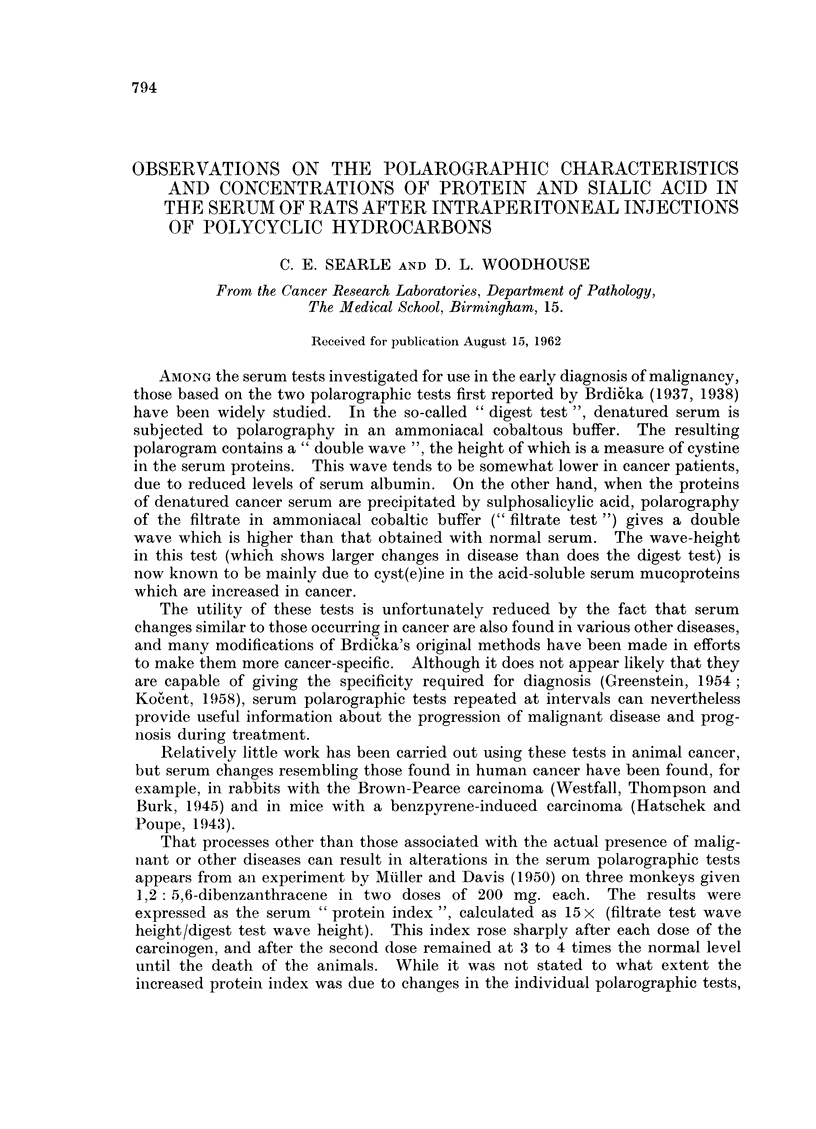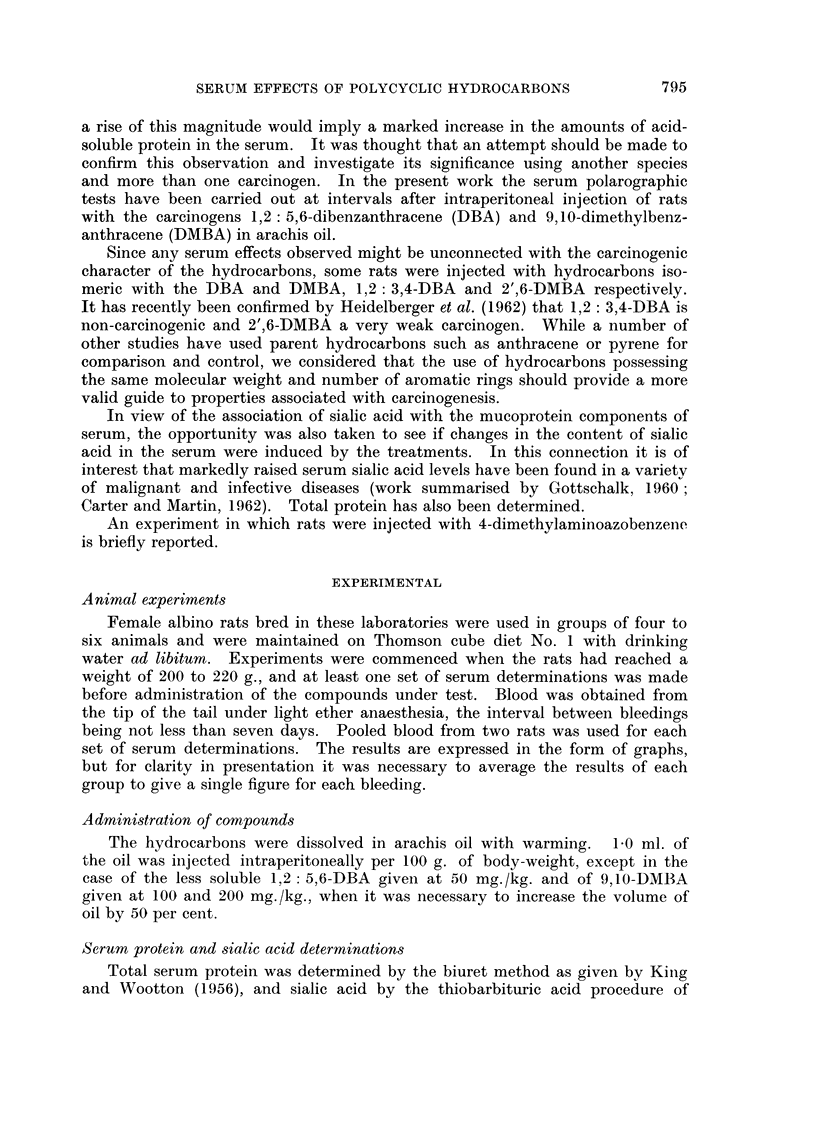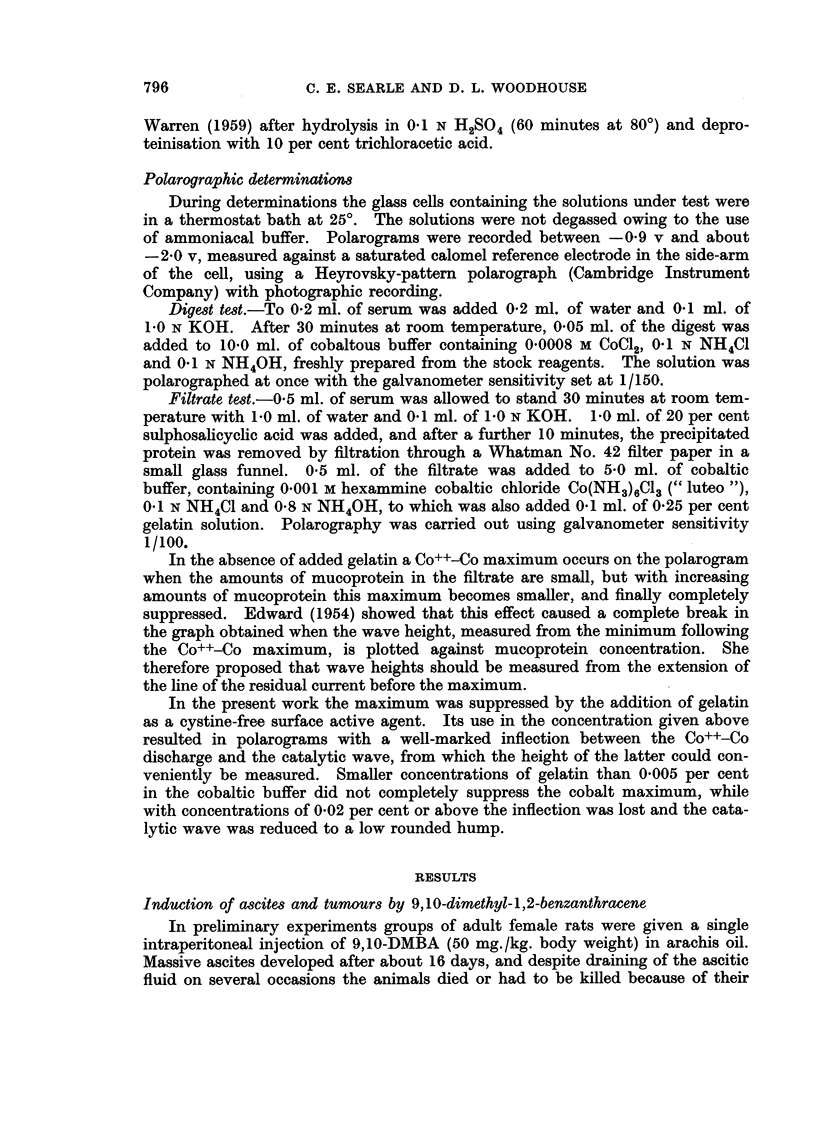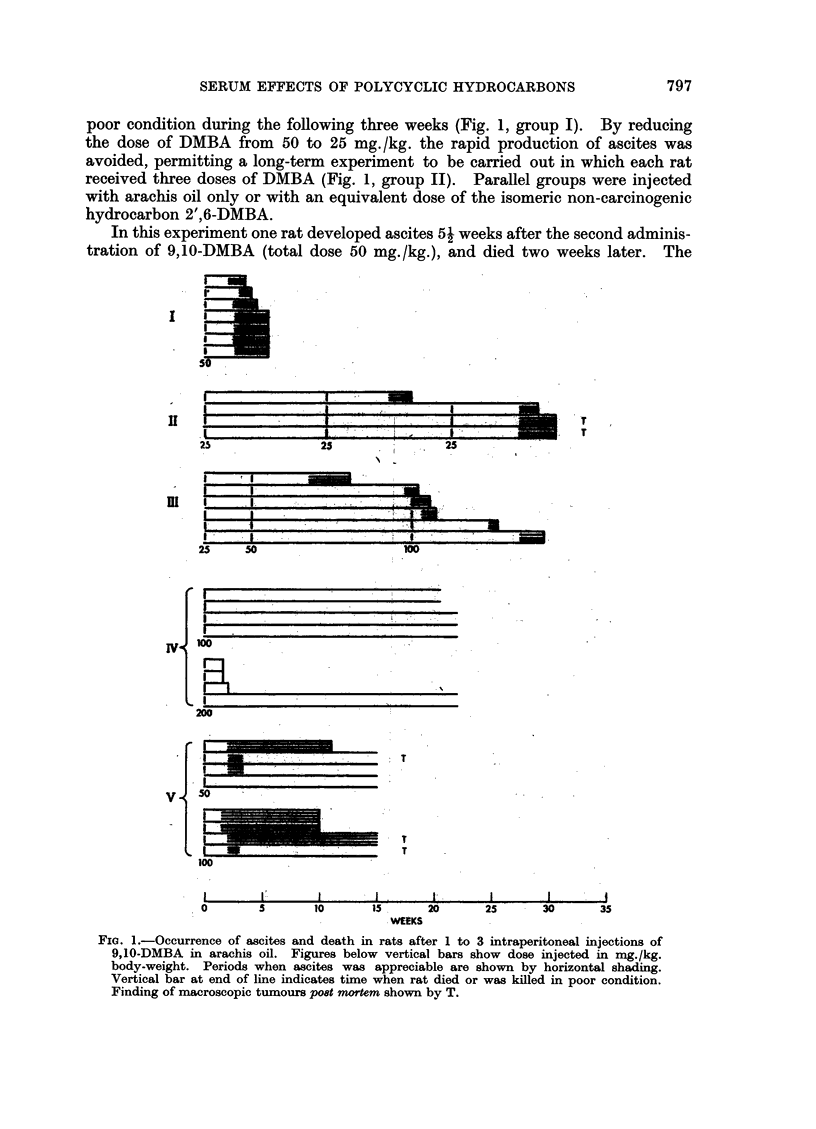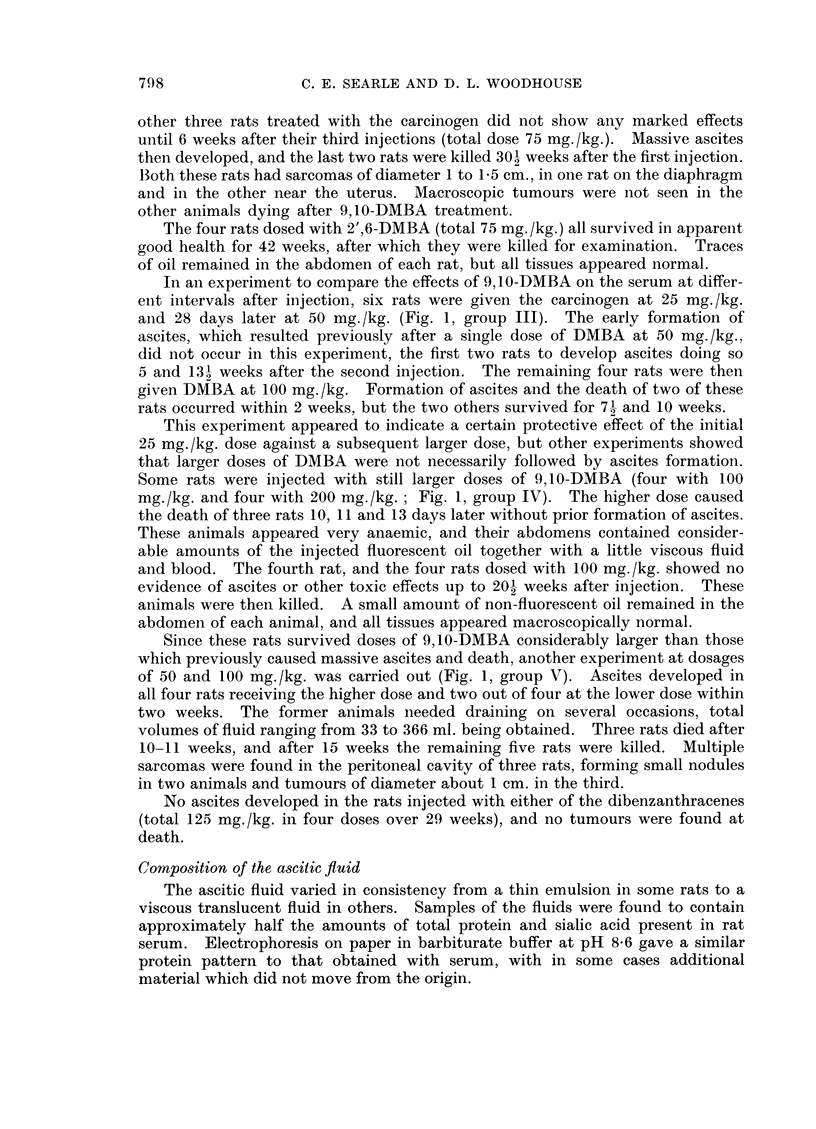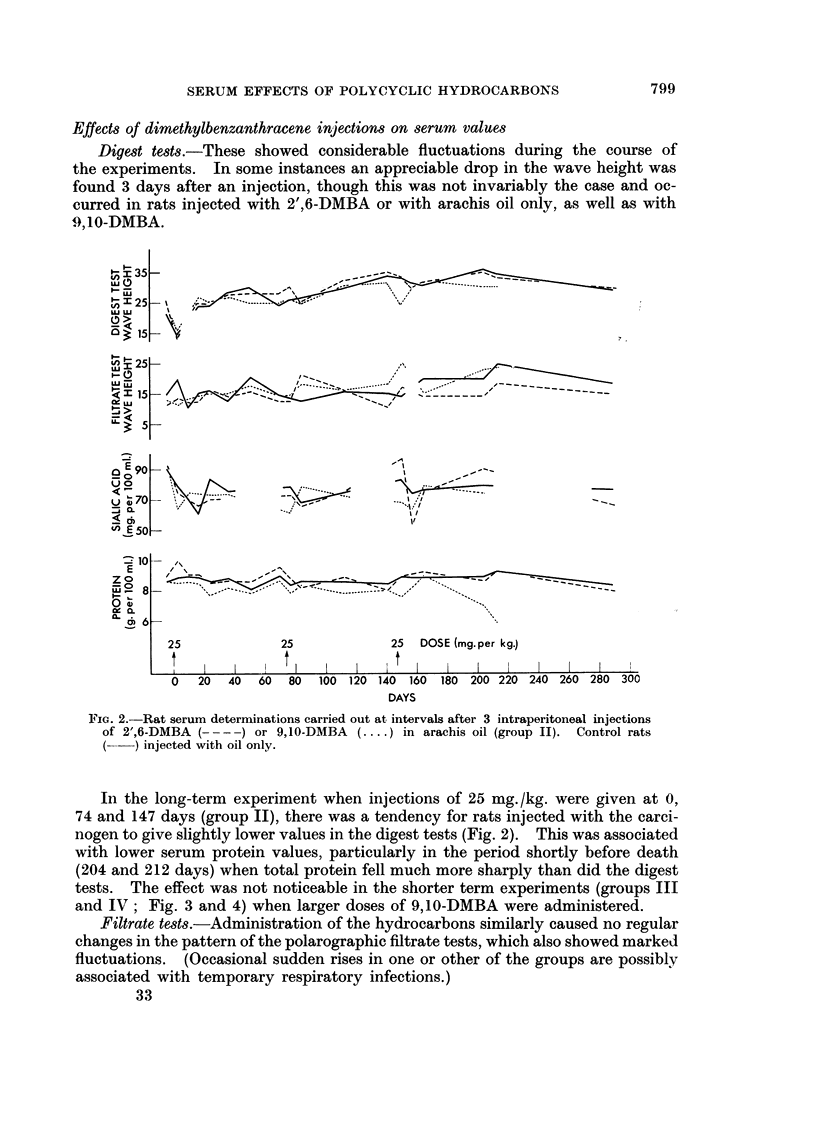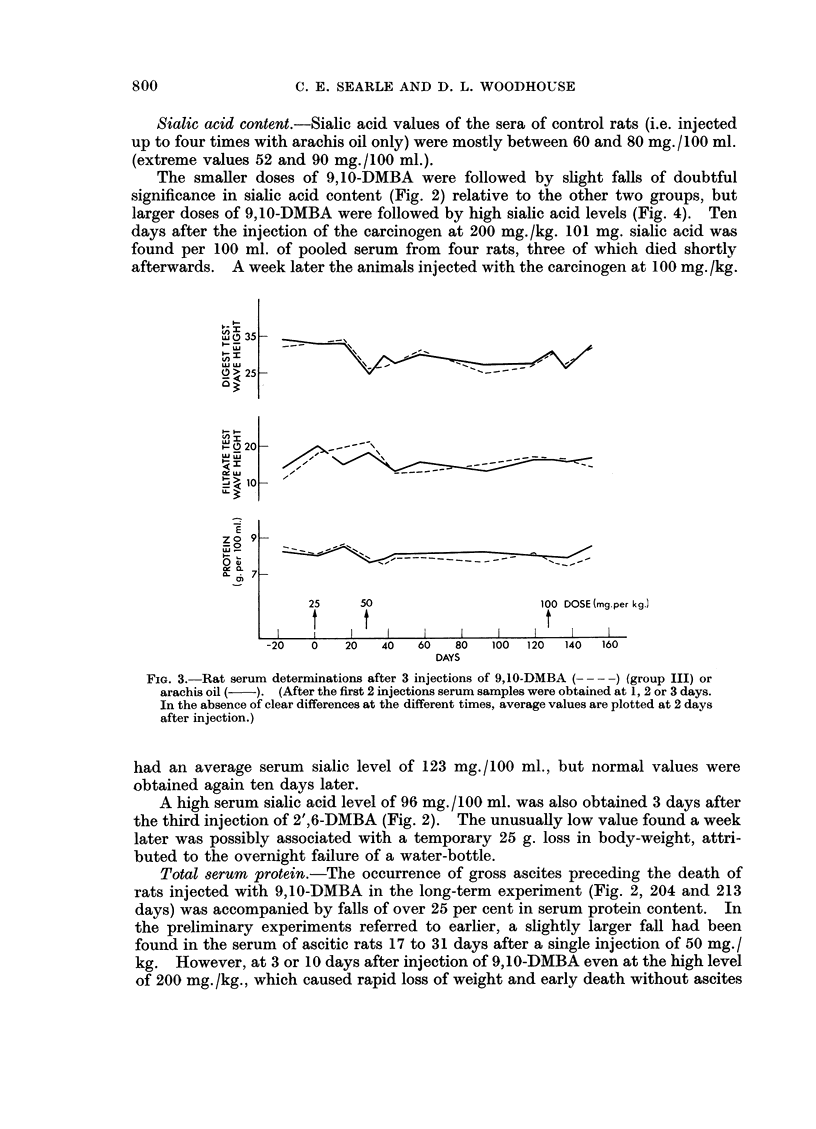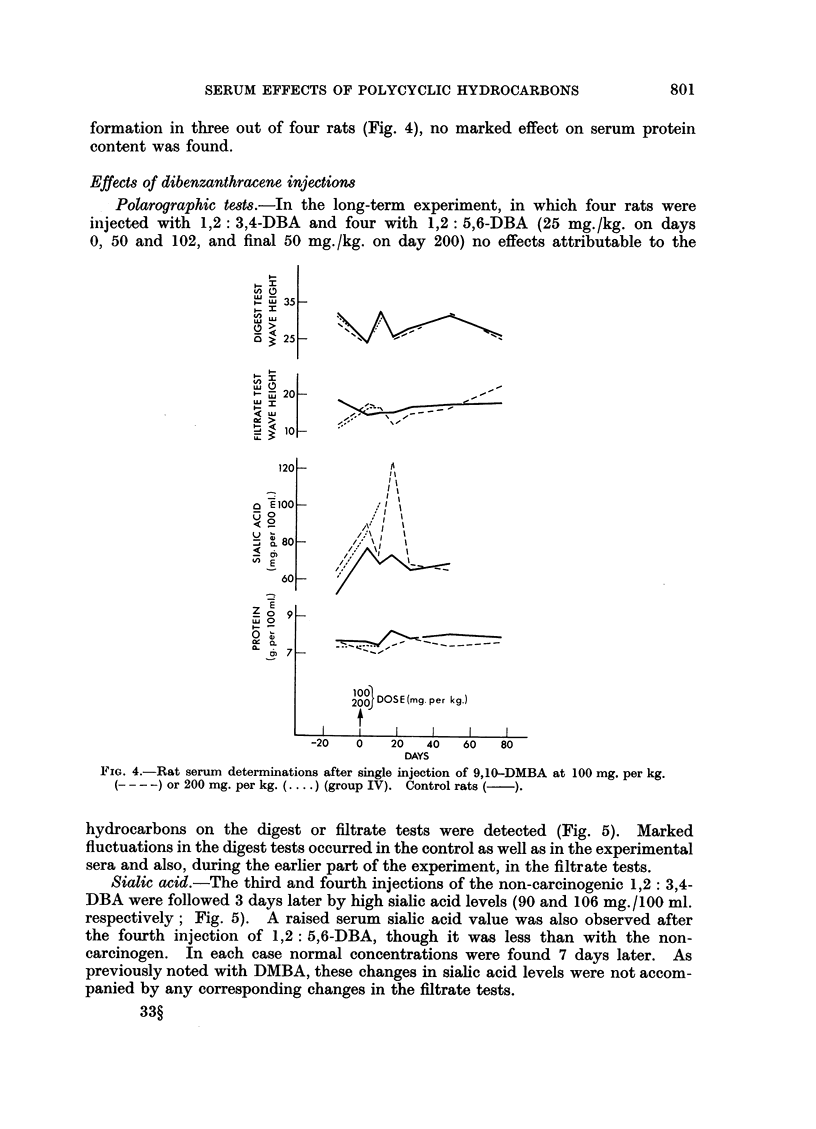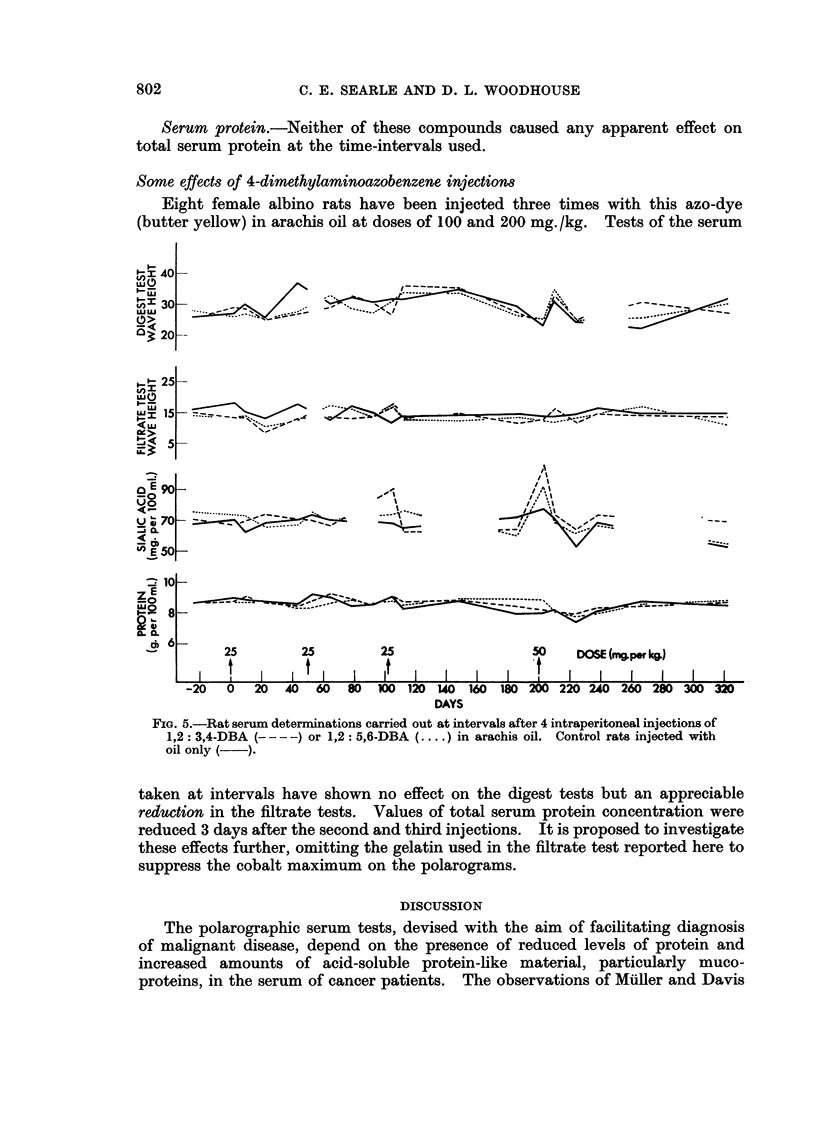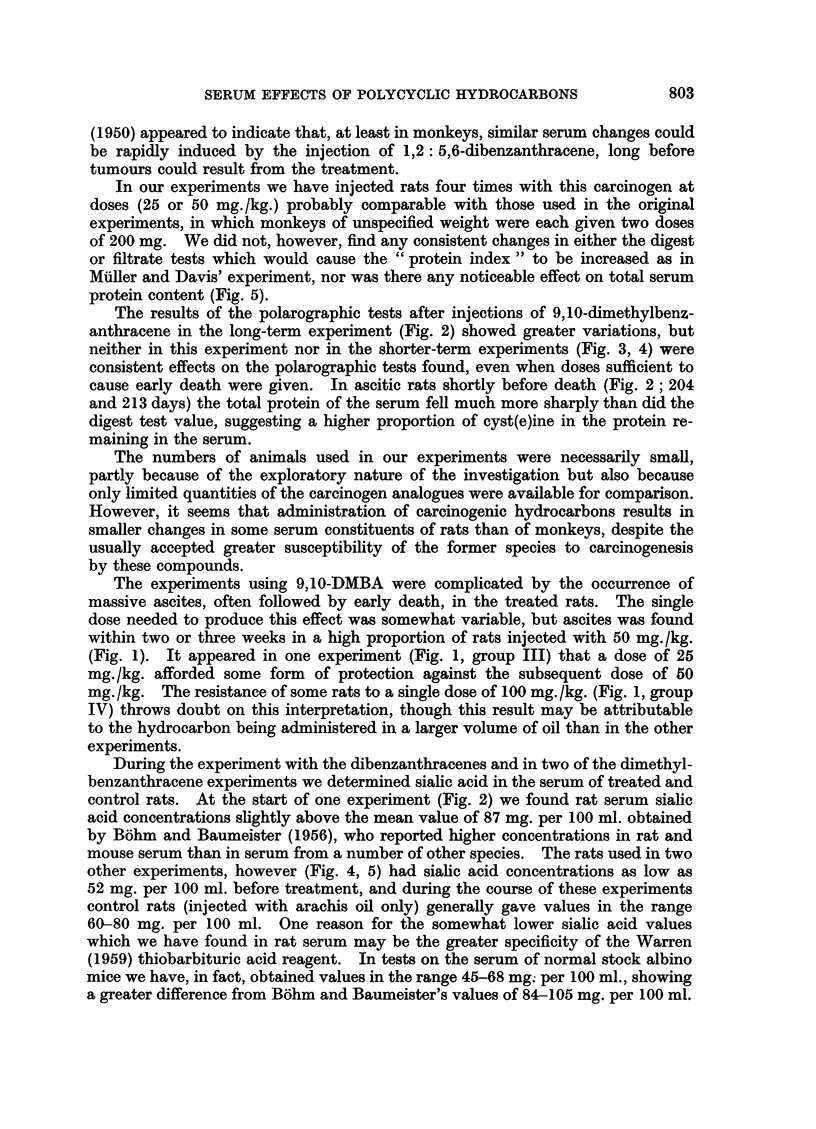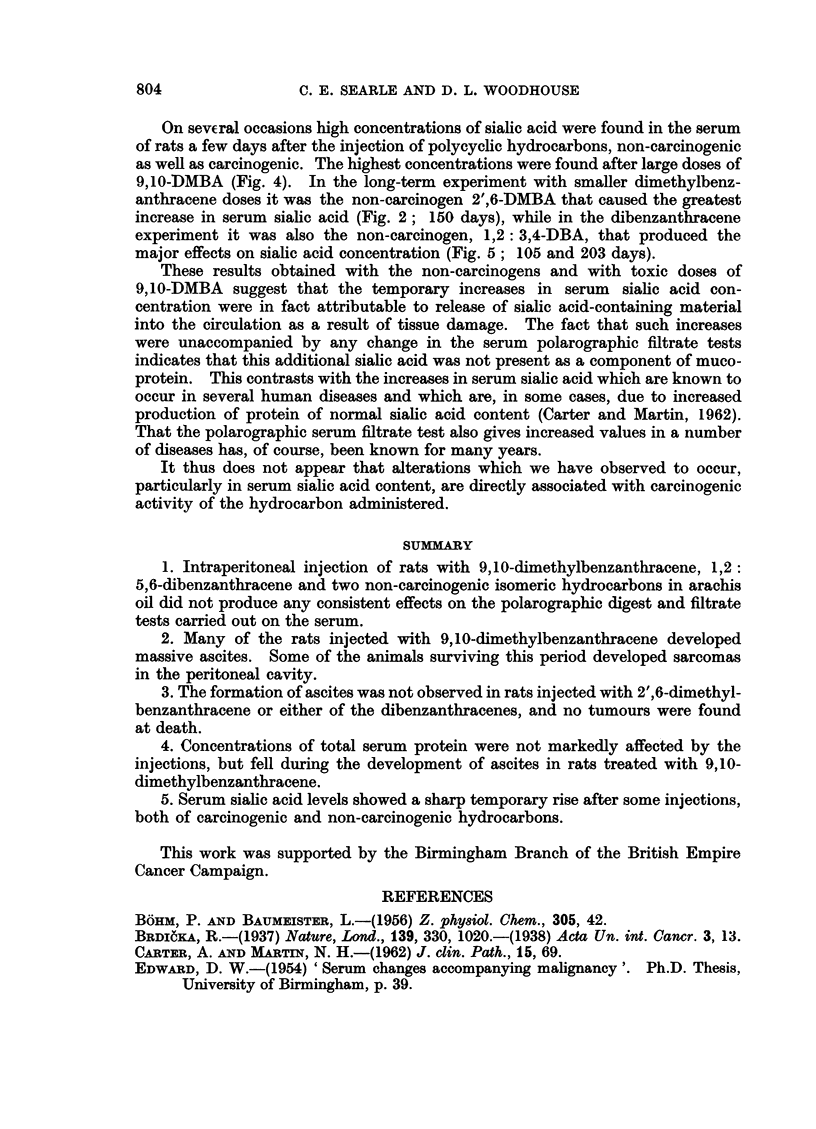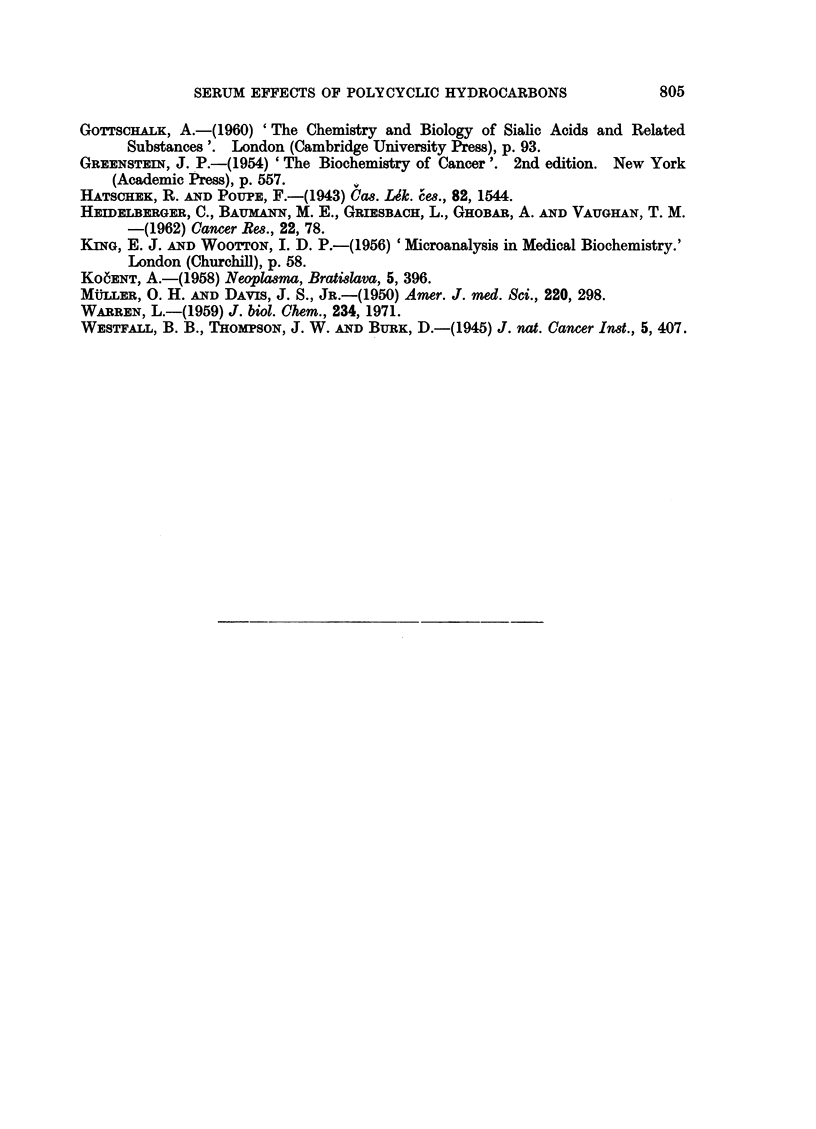# Observations on the Polarographic Characteristics and Concentrations of Protein and Sialic Acid in the Serum of Rats After Intraperitoneal Injections of Polycyclic Hydrocarbons

**DOI:** 10.1038/bjc.1962.92

**Published:** 1962-12

**Authors:** C. E. Searle, D. L. Woodhouse


### OBSERVATIONS ON THE POLAROGRAPHIC CHARACTERISTICS AND CONCENTRATIONS OF PROTEIN AND SIALIC ACID IN THE SERUM OF RATS AFTER INTRAPERITONEAL INJECTIONS OF POLYCYCLIC HYDROCARBONS

C. E. SEARLE AND D. L. WOODHOUSE

*From the Cancer Research Laboratories, Department of Pathology, The Medical School, Birmingham, 15.*

Received for publication August 15, 1962

AMONG the serum tests investigated for use in the early diagnosis of malignancy, those based on the two polarographic tests first reported by Brdička (1937, 1938) have been widely studied. In the so-called " digest test ", denatured serum is subjected to polarography in an ammoniacal cobaltous buffer. The resulting polarogram contains a " double wave ", the height of which is a measure of cystine in the serum proteins. This wave tends to be somewhat lower in cancer patients, due to reduced levels of serum albumin. On the other hand, when the proteins of denatured cancer serum are precipitated by sulphosalicylic acid, polarography of the filtrate in ammoniacal cobaltic buffer (" filtrate test ") gives a double wave which is higher than that obtained with normal serum. The wave-height in this test (which shows larger changes in disease than does the digest test) is now known to be mainly due to cyst(e)ine in the acid-soluble serum mucoproteins which are increased in cancer.

The utility of these tests is unfortunately reduced by the fact that serum changes similar to those occurring in cancer are also found in various other diseases, and many modifications of Brdička's original methods have been made in efforts to make them more cancer-specific. Although it does not appear likely that they are capable of giving the specificity required for diagnosis (Greenstein, 1954; Kočent, 1958), serum polarographic tests repeated at intervals can nevertheless provide useful information about the progression of malignant disease and prognosis during treatment.

Relatively little work has been carried out using these tests in animal cancer, but serum changes resembling those found in human cancer have been found, for example, in rabbits with the Brown-Pearce carcinoma (Westfall, Thompson and Burk, 1945) and in mice with a benzpyrene-induced carcinoma (Hatschek and Poupe, 1943).

That processes other than those associated with the actual presence of malignant or other diseases can result in alterations in the serum polarographic tests appears from an experiment by Müller and Davis (1950) on three monkeys given 1,2 : 5,6-dibenzanthracene in two doses of 200 mg. each. The results were expressed as the serum " protein index ", calculated as 15 $\times$ (filtrate test wave height/digest test wave height). This index rose sharply after each dose of the carcinogen, and after the second dose remained at 3 to 4 times the normal level until the death of the animals. While it was not stated to what extent the increased protein index was due to changes in the individual polarographic tests, a rise of this magnitude would imply a marked increase in the amounts of acid-soluble protein in the serum. It was thought that an attempt should be made to confirm this observation and investigate its significance using another species and more than one carcinogen. In the present work the serum polarographic tests have been carried out at intervals after intraperitoneal injection of rats with the carcinogens 1,2 : 5,6-dibenzanthracene (DBA) and 9,10-dimethylbenz-anthracene (DMBA) in arachis oil.

Since any serum effects observed might be unconnected with the carcinogenic character of the hydrocarbons, some rats were injected with hydrocarbons isomeric with the DBA and DMBA, 1,2 : 3,4-DBA and 2′,6-DMBA respectively. It has recently been confirmed by Heidelberger *et al.* (1962) that 1,2 : 3,4-DBA is non-carcinogenic and 2′,6-DMBA a very weak carcinogen. While a number of other studies have used parent hydrocarbons such as anthracene or pyrene for comparison and control, we considered that the use of hydrocarbons possessing the same molecular weight and number of aromatic rings should provide a more valid guide to properties associated with carcinogenesis.

In view of the association of sialic acid with the mucoprotein components of serum, the opportunity was also taken to see if changes in the content of sialic acid in the serum were induced by the treatments. In this connection it is of interest that markedly raised serum sialic acid levels have been found in a variety of malignant and infective diseases (work summarised by Gottschalk, 1960; Carter and Martin, 1962). Total protein has also been determined.

An experiment in which rats were injected with 4-dimethylaminoazobenzene is briefly reported.

### EXPERIMENTAL

### *Animal experiments*

Female albino rats bred in these laboratories were used in groups of four to six animals and were maintained on Thomson cube diet No. 1 with drinking water *ad libitum*. Experiments were commenced when the rats had reached a weight of 200 to 220 g., and at least one set of serum determinations was made before administration of the compounds under test. Blood was obtained from the tip of the tail under light ether anaesthesia, the interval between bleedings being not less than seven days. Pooled blood from two rats was used for each set of serum determinations. The results are expressed in the form of graphs, but for clarity in presentation it was necessary to average the results of each group to give a single figure for each bleeding.

### *Administration of compounds*

The hydrocarbons were dissolved in arachis oil with warming. 1·0 ml. of the oil was injected intraperitoneally per 100 g. of body-weight, except in the case of the less soluble 1,2 : 5,6-DBA given at 50 mg./kg. and of 9,10-DMBA given at 100 and 200 mg./kg., when it was necessary to increase the volume of oil by 50 per cent.

### *Serum protein and sialic acid determinations*

Total serum protein was determined by the biuret method as given by King and Wootton (1956), and sialic acid by the thiobarbituric acid procedure of

Warren (1959) after hydrolysis in 0·1 N $H_2SO_4$ (60 minutes at 80°) and deproteinisation with 10 per cent trichloracetic acid.

*Polarographic determinations*

During determinations the glass cells containing the solutions under test were in a thermostat bath at 25°. The solutions were not degassed owing to the use of ammoniacal buffer. Polarograms were recorded between —0·9 v and about —2·0 v, measured against a saturated calomel reference electrode in the side-arm of the cell, using a Heyrovsky-pattern polarograph (Cambridge Instrument Company) with photographic recording.

*Digest test.*—To 0·2 ml. of serum was added 0·2 ml. of water and 0·1 ml. of 1·0 N KOH. After 30 minutes at room temperature, 0·05 ml. of the digest was added to 10·0 ml. of cobaltous buffer containing 0·0008 M $CoCl_2$, 0·1 N $NH_4Cl$ and 0·1 N $NH_4OH$, freshly prepared from the stock reagents. The solution was polarographed at once with the galvanometer sensitivity set at 1/150.

*Filtrate test.*—0·5 ml. of serum was allowed to stand 30 minutes at room temperature with 1·0 ml. of water and 0·1 ml. of 1·0 N KOH. 1·0 ml. of 20 per cent sulphosalicyclic acid was added, and after a further 10 minutes, the precipitated protein was removed by filtration through a Whatman No. 42 filter paper in a small glass funnel. 0·5 ml. of the filtrate was added to 5·0 ml. of cobaltic buffer, containing 0·001 M hexammine cobaltic chloride $Co(NH_3)_6Cl_3$ (" luteo "), 0·1 N $NH_4Cl$ and 0·8 N $NH_4OH$, to which was also added 0·1 ml. of 0·25 per cent gelatin solution. Polarography was carried out using galvanometer sensitivity 1/100.

In the absence of added gelatin a $Co^{++}$–Co maximum occurs on the polarogram when the amounts of mucoprotein in the filtrate are small, but with increasing amounts of mucoprotein this maximum becomes smaller, and finally completely suppressed. Edward (1954) showed that this effect caused a complete break in the graph obtained when the wave height, measured from the minimum following the $Co^{++}$–Co maximum, is plotted against mucoprotein concentration. She therefore proposed that wave heights should be measured from the extension of the line of the residual current before the maximum.

In the present work the maximum was suppressed by the addition of gelatin as a cystine-free surface active agent. Its use in the concentration given above resulted in polarograms with a well-marked inflection between the $Co^{++}$–Co discharge and the catalytic wave, from which the height of the latter could conveniently be measured. Smaller concentrations of gelatin than 0·005 per cent in the cobaltic buffer did not completely suppress the cobalt maximum, while with concentrations of 0·02 per cent or above the inflection was lost and the catalytic wave was reduced to a low rounded hump.

### RESULTS

*Induction of ascites and tumours by* 9,10-*dimethyl*-1,2-*benzanthracene*

In preliminary experiments groups of adult female rats were given a single intraperitoneal injection of 9,10-DMBA (50 mg./kg. body weight) in arachis oil. Massive ascites developed after about 16 days, and despite draining of the ascitic fluid on several occasions the animals died or had to be killed because of their poor condition during the following three weeks (Fig. 1, group I). By reducing the dose of DMBA from 50 to 25 mg./kg. the rapid production of ascites was avoided, permitting a long-term experiment to be carried out in which each rat received three doses of DMBA (Fig. 1, group II). Parallel groups were injected with arachis oil only or with an equivalent dose of the isomeric non-carcinogenic hydrocarbon 2′,6-DMBA.

In this experiment one rat developed ascites $5\frac{1}{2}$ weeks after the second administration of 9,10-DMBA (total dose 50 mg./kg.), and died two weeks later. The

FIG. 1.—Occurrence of ascites and death in rats after 1 to 3 intraperitoneal injections of 9,10-DMBA in arachis oil. Figures below vertical bars show dose injected in mg./kg. body-weight. Periods when ascites was appreciable are shown by horizontal shading. Vertical bar at end of line indicates time when rat died or was killed in poor condition. Finding of macroscopic tumours *post mortem* shown by T.

other three rats treated with the carcinogen did not show any marked effects until 6 weeks after their third injections (total dose 75 mg./kg.). Massive ascites then developed, and the last two rats were killed 30½ weeks after the first injection. Both these rats had sarcomas of diameter 1 to 1·5 cm., in one rat on the diaphragm and in the other near the uterus. Macroscopic tumours were not seen in the other animals dying after 9,10-DMBA treatment.

The four rats dosed with 2′,6-DMBA (total 75 mg./kg.) all survived in apparent good health for 42 weeks, after which they were killed for examination. Traces of oil remained in the abdomen of each rat, but all tissues appeared normal.

In an experiment to compare the effects of 9,10-DMBA on the serum at different intervals after injection, six rats were given the carcinogen at 25 mg./kg. and 28 days later at 50 mg./kg. (Fig. 1, group III). The early formation of ascites, which resulted previously after a single dose of DMBA at 50 mg./kg., did not occur in this experiment, the first two rats to develop ascites doing so 5 and 13½ weeks after the second injection. The remaining four rats were then given DMBA at 100 mg./kg. Formation of ascites and the death of two of these rats occurred within 2 weeks, but the two others survived for 7½ and 10 weeks.

This experiment appeared to indicate a certain protective effect of the initial 25 mg./kg. dose against a subsequent larger dose, but other experiments showed that larger doses of DMBA were not necessarily followed by ascites formation. Some rats were injected with still larger doses of 9,10-DMBA (four with 100 mg./kg. and four with 200 mg./kg. ; Fig. 1, group IV). The higher dose caused the death of three rats 10, 11 and 13 days later without prior formation of ascites. These animals appeared very anaemic, and their abdomens contained considerable amounts of the injected fluorescent oil together with a little viscous fluid and blood. The fourth rat, and the four rats dosed with 100 mg./kg. showed no evidence of ascites or other toxic effects up to 20½ weeks after injection. These animals were then killed. A small amount of non-fluorescent oil remained in the abdomen of each animal, and all tissues appeared macroscopically normal.

Since these rats survived doses of 9,10-DMBA considerably larger than those which previously caused massive ascites and death, another experiment at dosages of 50 and 100 mg./kg. was carried out (Fig. 1, group V). Ascites developed in all four rats receiving the higher dose and two out of four at the lower dose within two weeks. The former animals needed draining on several occasions, total volumes of fluid ranging from 33 to 366 ml. being obtained. Three rats died after 10–11 weeks, and after 15 weeks the remaining five rats were killed. Multiple sarcomas were found in the peritoneal cavity of three rats, forming small nodules in two animals and tumours of diameter about 1 cm. in the third.

No ascites developed in the rats injected with either of the dibenzanthracenes (total 125 mg./kg. in four doses over 29 weeks), and no tumours were found at death.

*Composition of the ascitic fluid*

The ascitic fluid varied in consistency from a thin emulsion in some rats to a viscous translucent fluid in others. Samples of the fluids were found to contain approximately half the amounts of total protein and sialic acid present in rat serum. Electrophoresis on paper in barbiturate buffer at pH 8·6 gave a similar protein pattern to that obtained with serum, with in some cases additional material which did not move from the origin.

*Effects of dimethylbenzanthracene injections on serum values*

*Digest tests.*—These showed considerable fluctuations during the course of the experiments. In some instances an appreciable drop in the wave height was found 3 days after an injection, though this was not invariably the case and occurred in rats injected with 2′,6-DMBA or with arachis oil only, as well as with 9,10-DMBA.

FIG. 2.—Rat serum determinations carried out at intervals after 3 intraperitoneal injections of 2′,6-DMBA (– – – –) or 9,10-DMBA (. . . .) in arachis oil (group II). Control rats (———) injected with oil only.

In the long-term experiment when injections of 25 mg./kg. were given at 0, 74 and 147 days (group II), there was a tendency for rats injected with the carcinogen to give slightly lower values in the digest tests (Fig. 2). This was associated with lower serum protein values, particularly in the period shortly before death (204 and 212 days) when total protein fell much more sharply than did the digest tests. The effect was not noticeable in the shorter term experiments (groups III and IV ; Fig. 3 and 4) when larger doses of 9,10-DMBA were administered.

*Filtrate tests.*—Administration of the hydrocarbons similarly caused no regular changes in the pattern of the polarographic filtrate tests, which also showed marked fluctuations. (Occasional sudden rises in one or other of the groups are possibly associated with temporary respiratory infections.)

*Sialic acid content.*—Sialic acid values of the sera of control rats (i.e. injected up to four times with arachis oil only) were mostly between 60 and 80 mg./100 ml. (extreme values 52 and 90 mg./100 ml.).

The smaller doses of 9,10-DMBA were followed by slight falls of doubtful significance in sialic acid content (Fig. 2) relative to the other two groups, but larger doses of 9,10-DMBA were followed by high sialic acid levels (Fig. 4). Ten days after the injection of the carcinogen at 200 mg./kg. 101 mg. sialic acid was found per 100 ml. of pooled serum from four rats, three of which died shortly afterwards. A week later the animals injected with the carcinogen at 100 mg./kg.

FIG. 3.—Rat serum determinations after 3 injections of 9,10-DMBA (- - - -) (group III) or arachis oil (——). (After the first 2 injections serum samples were obtained at 1, 2 or 3 days. In the absence of clear differences at the different times, average values are plotted at 2 days after injection.)

had an average serum sialic level of 123 mg./100 ml., but normal values were obtained again ten days later.

A high serum sialic acid level of 96 mg./100 ml. was also obtained 3 days after the third injection of 2′,6-DMBA (Fig. 2). The unusually low value found a week later was possibly associated with a temporary 25 g. loss in body-weight, attributed to the overnight failure of a water-bottle.

*Total serum protein.*—The occurrence of gross ascites preceding the death of rats injected with 9,10-DMBA in the long-term experiment (Fig. 2, 204 and 213 days) was accompanied by falls of over 25 per cent in serum protein content. In the preliminary experiments referred to earlier, a slightly larger fall had been found in the serum of ascitic rats 17 to 31 days after a single injection of 50 mg./kg. However, at 3 or 10 days after injection of 9,10-DMBA even at the high level of 200 mg./kg., which caused rapid loss of weight and early death without ascites formation in three out of four rats (Fig. 4), no marked effect on serum protein content was found.

*Effects of dibenzanthracene injections*

*Polarographic tests.*—In the long-term experiment, in which four rats were injected with 1,2 : 3,4-DBA and four with 1,2 : 5,6-DBA (25 mg./kg. on days 0, 50 and 102, and final 50 mg./kg. on day 200) no effects attributable to the

FIG. 4.—Rat serum determinations after single injection of 9,10-DMBA at 100 mg. per kg. (- - - -) or 200 mg. per kg. (. . . .) (group IV). Control rats (——).

hydrocarbons on the digest or filtrate tests were detected (Fig. 5). Marked fluctuations in the digest tests occurred in the control as well as in the experimental sera and also, during the earlier part of the experiment, in the filtrate tests.

*Sialic acid.*—The third and fourth injections of the non-carcinogenic 1,2 : 3,4-DBA were followed 3 days later by high sialic acid levels (90 and 106 mg./100 ml. respectively ; Fig. 5). A raised serum sialic acid value was also observed after the fourth injection of 1,2 : 5,6-DBA, though it was less than with the non-carcinogen. In each case normal concentrations were found 7 days later. As previously noted with DMBA, these changes in sialic acid levels were not accompanied by any corresponding changes in the filtrate tests.

*Serum protein.*—Neither of these compounds caused any apparent effect on total serum protein at the time-intervals used.

*Some effects of 4-dimethylaminoazobenzene injections*

Eight female albino rats have been injected three times with this azo-dye (butter yellow) in arachis oil at doses of 100 and 200 mg./kg. Tests of the serum

(1950) appeared to indicate that, at least in monkeys, similar serum changes could be rapidly induced by the injection of 1,2 : 5,6-dibenzanthracene, long before tumours could result from the treatment.

In our experiments we have injected rats four times with this carcinogen at doses (25 or 50 mg./kg.) probably comparable with those used in the original experiments, in which monkeys of unspecified weight were each given two doses of 200 mg. We did not, however, find any consistent changes in either the digest or filtrate tests which would cause the " protein index " to be increased as in Müller and Davis' experiment, nor was there any noticeable effect on total serum protein content (Fig. 5).

The results of the polarographic tests after injections of 9,10-dimethylbenzanthracene in the long-term experiment (Fig. 2) showed greater variations, but neither in this experiment nor in the shorter-term experiments (Fig. 3, 4) were consistent effects on the polarographic tests found, even when doses sufficient to cause early death were given. In ascitic rats shortly before death (Fig. 2 ; 204 and 213 days) the total protein of the serum fell much more sharply than did the digest test value, suggesting a higher proportion of cyst(e)ine in the protein remaining in the serum.

The numbers of animals used in our experiments were necessarily small, partly because of the exploratory nature of the investigation but also because only limited quantities of the carcinogen analogues were available for comparison. However, it seems that administration of carcinogenic hydrocarbons results in smaller changes in some serum constituents of rats than of monkeys, despite the usually accepted greater susceptibility of the former species to carcinogenesis by these compounds.

The experiments using 9,10-DMBA were complicated by the occurrence of massive ascites, often followed by early death, in the treated rats. The single dose needed to produce this effect was somewhat variable, but ascites was found within two or three weeks in a high proportion of rats injected with 50 mg./kg. (Fig. 1). It appeared in one experiment (Fig. 1, group III) that a dose of 25 mg./kg. afforded some form of protection against the subsequent dose of 50 mg./kg. The resistance of some rats to a single dose of 100 mg./kg. (Fig. 1, group IV) throws doubt on this interpretation, though this result may be attributable to the hydrocarbon being administered in a larger volume of oil than in the other experiments.

During the experiment with the dibenzanthracenes and in two of the dimethylbenzanthracene experiments we determined sialic acid in the serum of treated and control rats. At the start of one experiment (Fig. 2) we found rat serum sialic acid concentrations slightly above the mean value of 87 mg. per 100 ml. obtained by Böhm and Baumeister (1956), who reported higher concentrations in rat and mouse serum than in serum from a number of other species. The rats used in two other experiments, however (Fig. 4, 5) had sialic acid concentrations as low as 52 mg. per 100 ml. before treatment, and during the course of these experiments control rats (injected with arachis oil only) generally gave values in the range 60–80 mg. per 100 ml. One reason for the somewhat lower sialic acid values which we have found in rat serum may be the greater specificity of the Warren (1959) thiobarbituric acid reagent. In tests on the serum of normal stock albino mice we have, in fact, obtained values in the range 45–68 mg. per 100 ml., showing a greater difference from Böhm and Baumeister's values of 84–105 mg. per 100 ml.

On several occasions high concentrations of sialic acid were found in the serum of rats a few days after the injection of polycyclic hydrocarbons, non-carcinogenic as well as carcinogenic. The highest concentrations were found after large doses of 9,10-DMBA (Fig. 4). In the long-term experiment with smaller dimethylbenzanthracene doses it was the non-carcinogen 2′,6-DMBA that caused the greatest increase in serum sialic acid (Fig. 2 ; 150 days), while in the dibenzanthracene experiment it was also the non-carcinogen, 1,2 : 3,4-DBA, that produced the major effects on sialic acid concentration (Fig. 5 ; 105 and 203 days).

These results obtained with the non-carcinogens and with toxic doses of 9,10-DMBA suggest that the temporary increases in serum sialic acid concentration were in fact attributable to release of sialic acid-containing material into the circulation as a result of tissue damage. The fact that such increases were unaccompanied by any change in the serum polarographic filtrate tests indicates that this additional sialic acid was not present as a component of mucoprotein. This contrasts with the increases in serum sialic acid which are known to occur in several human diseases and which are, in some cases, due to increased production of protein of normal sialic acid content (Carter and Martin, 1962). That the polarographic serum filtrate test also gives increased values in a number of diseases has, of course, been known for many years.

It thus does not appear that alterations which we have observed to occur, particularly in serum sialic acid content, are directly associated with carcinogenic activity of the hydrocarbon administered.

### SUMMARY

1. Intraperitoneal injection of rats with 9,10-dimethylbenzanthracene, 1,2 : 5,6-dibenzanthracene and two non-carcinogenic isomeric hydrocarbons in arachis oil did not produce any consistent effects on the polarographic digest and filtrate tests carried out on the serum.

2. Many of the rats injected with 9,10-dimethylbenzanthracene developed massive ascites. Some of the animals surviving this period developed sarcomas in the peritoneal cavity.

3. The formation of ascites was not observed in rats injected with 2′,6-dimethylbenzanthracene or either of the dibenzanthracenes, and no tumours were found at death.

4. Concentrations of total serum protein were not markedly affected by the injections, but fell during the development of ascites in rats treated with 9,10-dimethylbenzanthracene.

5. Serum sialic acid levels showed a sharp temporary rise after some injections, both of carcinogenic and non-carcinogenic hydrocarbons.

This work was supported by the Birmingham Branch of the British Empire Cancer Campaign.

### REFERENCES

Böhm, P. and Baumeister, L.—(1956) *Z. physiol. Chem.*, **305**, 42.
Brdička, R.—(1937) *Nature, Lond.*, **139**, 330, 1020.—(1938) *Acta Un. int. Cancr.* **3**, 13.
Carter, A. and Martin, N. H.—(1962) *J. clin. Path.*, **15**, 69.
Edward, D. W.—(1954) ‘ Serum changes accompanying malignancy ’. Ph.D. Thesis, University of Birmingham, p. 39.

GOTTSCHALK, A.—(1960) 'The Chemistry and Biology of Sialic Acids and Related Substances'. London (Cambridge University Press), p. 93.
GREENSTEIN, J. P.—(1954) 'The Biochemistry of Cancer'. 2nd edition. New York (Academic Press), p. 557.
HATSCHEK, R. AND POUPE, F.—(1943) *Čas. Lék. čes.*, **82**, 1544.
HEIDELBERGER, C., BAUMANN, M. E., GRIESBACH, L., GHOBAR, A. AND VAUGHAN, T. M. —(1962) *Cancer Res.*, **22**, 78.
KING, E. J. AND WOOTTON, I. D. P.—(1956) 'Microanalysis in Medical Biochemistry.' London (Churchill), p. 58.
KOČENT, A.—(1958) *Neoplasma, Bratislava*, **5**, 396.
MÜLLER, O. H. AND DAVIS, J. S., JR.—(1950) *Amer. J. med. Sci.*, **220**, 298.
WARREN, L.—(1959) *J. biol. Chem.*, **234**, 1971.
WESTFALL, B. B., THOMPSON, J. W. AND BURK, D.—(1945) *J. nat. Cancer Inst.*, **5**, 407.